# Bilateral pulmonary embolism while receiving tranexamic acid: a case report

**DOI:** 10.1186/s13256-020-02545-z

**Published:** 2020-11-06

**Authors:** Ezekiel Oluwasayo Ijaopo, Ruth Oluwasolape Ijaopo, Sampson Adjei

**Affiliations:** 1 East Kent Hospitals University NHS Foundation Trust, William Harvey Hospital, Ashford, Kent UK; 2 University Hospitals Birmingham NHS Foundation Trust, Queen Elizabeth Hospital Birmingham, Birmingham, B15 2TH UK

**Keywords:** Pulmonary embolism, Venous thromboembolism, Tranexamic acid, Antifibrinolytic therapy, Case report

## Abstract

**Background:**

We present a case of a suspected tranexamic acid–related bilateral pulmonary embolism in a healthy and active middle-aged woman who was receiving tranexamic acid for menorrhagia with no other known significant risk factors for thromboembolism.

**Case presentation:**

A 46-year-old Asian woman who was usually fit and well with no remarkable past medical history except for menorrhagia of 1-year duration for which she was receiving tranexamic acid presented to our accident and emergency department with a 2-week history of intermittent pleuritic central chest pain. She was reviewed and discharged to home with a diagnosis of musculoskeletal pain on two hospital visits because she had no significant risk factors for thromboembolism and her workup investigation results for pulmonary embolism and other differential diagnoses were largely unremarkable. On her third visit to the emergency ambulatory clinic with recurring symptoms of pleuritic chest pain, a pulmonary computed tomographic angiogram confirmed bilateral subsegmental pulmonary embolism.

**Conclusion:**

This case report reinforces the possible increased risk of thromboembolism in patients receiving tranexamic acid.

## Introduction

Pulmonary embolism (PE) is a common medical condition and remains life-threatening despite advances in its diagnosis and treatment past few decades. The incidence of PE is estimated to be approximately 60 to 70 per 100,000 in the general population [1]. However, the true incidence is far more than what is reported, because PE remains one of the most commonly underdiagnosed medical problems. It is believed to be responsible for 100,000 deaths per year in the United States [2]. In Europe, cases of PE affect 6 to 20 per 10,000 people per year, and 7–11% of people with PE do not survive [3]. If untreated, mortality of acute PE is as high as 30%, whereas the death rate of diagnosed and treated PE is 8% [1]. The confirmation of acute PE diagnosis via lung imaging relies on the history, physical examination, and a high index of clinical suspicion [4]. Prompt diagnosis and treatment are therefore imperative to reduce the morbidity and mortality of PE. This case report provides increased awareness for clinicians on how a commonly prescribed tranexamic acid (TXA) can be a potential cause of PE, particularly in patients considered to have very low risk factors for venous thromboembolism (VTE).

## Case presentation

### Background

We present a case of a 46-year-old Asian woman who was usually fit and well except for a 1-year history of menorrhagia prior to her initial presentation in our emergency department (ED). Her menorrhagia was due to multiple fibroids diagnosed via transvaginal ultrasound of the pelvis in 2018, which showed a multifibroid uterus with normal-appearing ovaries and no obvious adnexal cysts/masses. She was then started on TXA (1 g three times daily as required) and mefenamic acid (500 mg three times daily as required) to be taken during her menstrual period to reduce excessive bleeding and pain, respectively. She claimed she did not have to take the TXA (and mefenamic acid) during all her menstrual periods, because she believed the TXA was not required on many occasions. She was physically healthy, of normal weight (body mass index of 22 kg/m^2^), never smoked cigarettes or drank alcohol, and had no previous history of DVT or PE. She also denied using any form of contraception and had no significant family history of clotting disorders or cancer, but she claimed her mother had type 2 diabetes mellitus and had died of myocardial infarction.

### First hospital visit (autumn 2019)

Our patient presented to our ED with a 2-week history of noncardiac-type central chest pain that was nonradiating, pleuritic, and intermittent with occasional shortness of breath on exertion. She had no history of diaphoresis, nausea, vomiting, cough, fever, or any infective symptoms. She had no history of recent long-distance journey or any other significant risk factors suggestive of VTE.

Except for a fast heart rate (119 beats/minute), her vital signs, including blood pressure and physical examination, were within normal limits. Her chest x-ray was normal, and her Electrocardiogram (ECG) showed no dynamic changes except for sinus tachycardia. Her D-dimer was marginally raised at 0.66 μg/ml (normal range, 0.05 to 0.50 μg/ml), whereas her cardiac troponin I finding was negative. Other routine blood test results, including electrolytes, complete blood count, inflammatory markers, and clotting screen, were within normal limits. She was diagnosed with possible anxiety/musculoskeletal pain and sent home with analgesics and a planned follow-up review of her symptoms in the emergency ambulatory clinic (EAC) after 1 week.

### Follow-up review

About 2 weeks after her initial presentation, the patient came back for follow-up review in the EAC as planned. She claimed she still experienced pleuritic chest pain on and off in addition to a new intermittent interscapular pain. A repeat D-dimer test result came back negative (0.35 μg/ml; normal range, 0.05 to 0.50 μg/ml). Likewise, results of her physical examination and recheck of her routine blood tests, including troponin I, clotting screen, and inflammatory markers, were all within normal limits. She was reassured and discharged to home after a (repeat) normal chest x-ray finding. She was informed that a computed tomographic (CT) pulmonary angiogram (CTPA) or ventilation/perfusion measurement was not required.

### Third visit

**Fig. 1:**
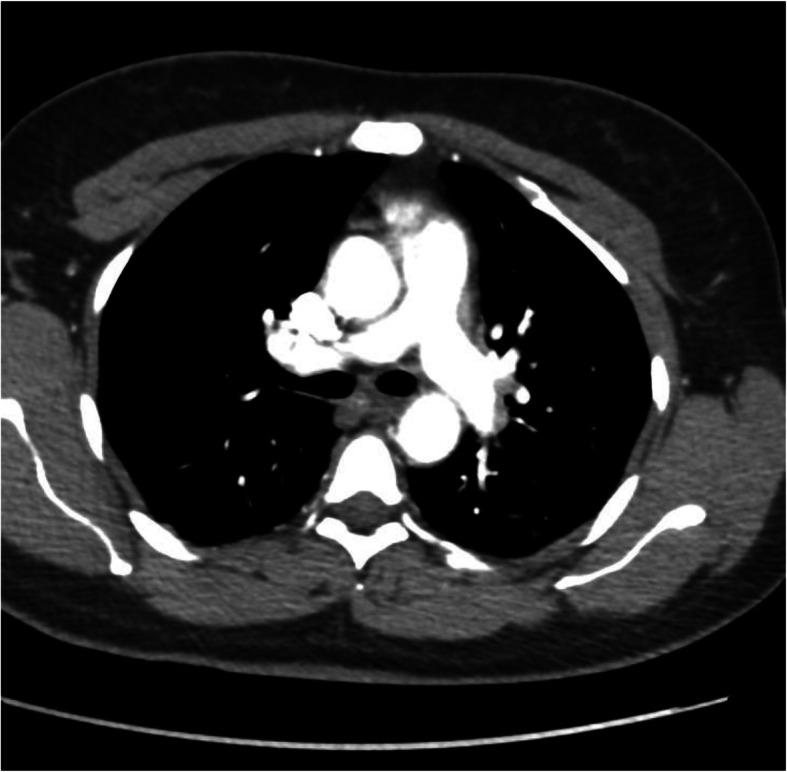
Left lower lobe subsegmental PE

**Fig. 2:**
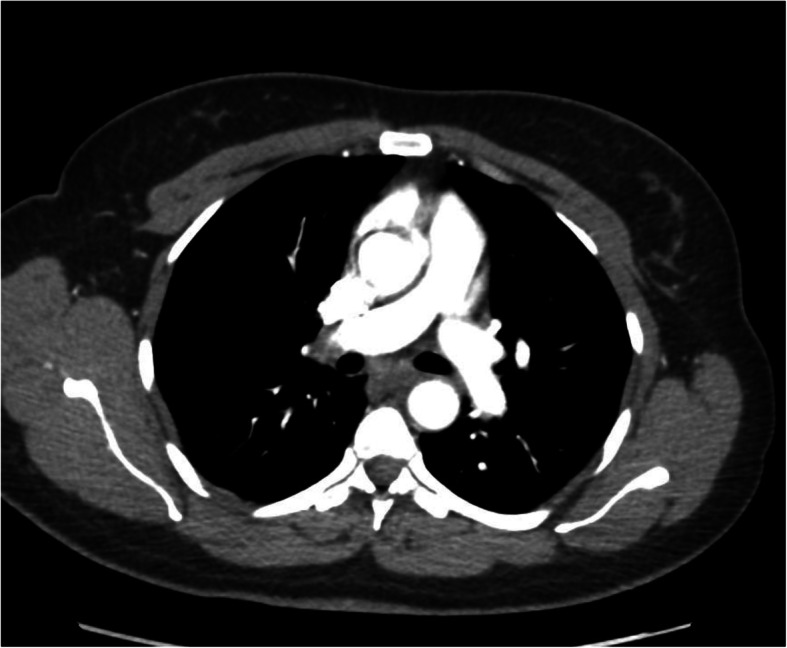
Right upper lobe subsegmental PE

About 2 months after the follow-up review, our patient re-presented to our ED with symptoms of pleuritic central chest pain and intermittent shortness of breath on moderate exertion. She claimed her symptoms were similar to her previous presentations. Further history was taken to exclude infection, cardiac-related problems, and common risk factors for PE, among other illnesses, but the findings were unremarkable. The patient said she last took her TXA for 2 days before the index presentation. Her physical examination results, including respiratory and cardiovascular examinations, were as usual within normal limits. Her vital signs were normal except for tachycardia (pulse rate of 113 beats/minute). Her blood workup showed slightly raised D-dimer (0.93 μg/ml), but other routine blood results for infection, thyroid function, electrolytes, clotting screen, complete blood count, and cardiac biomarkers were again all within normal limits. Her ECG showed sinus tachycardia, but her chest x-ray finding again was normal. Wells Score for PE was 4.5. We had a high suspicion to exclude PE in view of her symptoms and TXA use. So, a therapeutic dose of enoxaparin was started, and we placed an order for CTPA. The CTPA report 2 days later demonstrated filling defects in the distal subsegmental branches of the left lower and right upper segments that confirmed bilateral subsegmental PE (see Figs. 1 and 2).

### Treatment

Following the confirmation of PE diagnosis on the basis of imaging, our patient’s treatment dose of enoxaparin was changed to apixaban. The planned duration of treatment with apixaban was 3 months; however, this is usually subject to evaluation during patient follow-up in the anticoagulation clinic. Our patient was then advised to stop TXA and informed to use other painkillers, such as paracetamol and/or codeine phosphate, for pain control instead of mefenamic acid due to increased risk of bleeding caused by drug–drug interactions with apixaban.

### Follow-up and referrals

An outpatient CT scan of the patient’s abdomen and pelvis (CT-AP) was arranged and obtained within 2 weeks after PE diagnosis to rule out any occult malignancy. The CT-AP scan report finding was normal. The patient was subsequently referred for routine follow-up in the anticoagulation clinic within the hematology unit (as per our hospital policy). In the anticoagulation clinic, a patient with acute VTE would usually undergo further evaluation as may be necessary including workup for thrombophilia screen and a decision on duration of anticoagulation treatment is made.

After 1-month follow-up of the patient over the telephone, she claimed her pleuritic chest pain has improved significantly and her menorrhagia and menstrual pain remained stable. However, about 11 weeks into the treatment with apixaban, while the patient was under follow-up in the anticoagulation clinic, she was sent for a repeat CTPA due to new-onset cough and breathlessness on exertion together with a raised D-dimer of 0.76. The repeat CTPA scan report showed that the PE noted seen on the previous scan had resolved, and no evidence of a new PE was seen, but there was new consolidation in the right lung. She was treated accordingly with appropriate antibiotics with a good clinical response. Following the resolution of symptoms, the decision was then made in the anticoagulation clinic that thrombophilia screening was no longer indicated in the patient at that time.

## Discussion

TXA is a generally well tolerated, safe, and effective drug commonly used in the prevention and treatment of excessive bleeding caused by medical, surgical, and postsurgical conditions for which antifibrinolytic therapy is appropriate [5]. It works by directly blocking plasmin formation, displaces plasminogen from the fibrin surface, and has an anti-inflammatory action that occurs through the inhibition of plasmin-mediated activation of complement, monocytes, and neutrophils [6].

Although it has been reported that TXA is linked to the development of thromboembolic events, some studies have claimed it does not increase the risk of venous or arterial thrombotic complications [7–10]. In contrast, several other studies have presented concerns about the possible association of TXA with development of VTE [11–14]. A newly published TXA risk evaluation in combat casualties study in trauma patients with severe injuries who were prescribed TXA reported a 3% increased chance of VTE and 9.4% higher odds of PE [15].

In our patient, TXA appeared to be the likely cause of the PE because no other significant risk factor for PE was identified. The empirical association between PE and TXA in our patient was further buttressed by the negative finding of PE on repeat CTPA as well as the resolution of clinical symptoms after TXA was stopped. This latter statement probably explained why the Hematology team in anticoagulation clinic concluded that thrombophilia screen was no longer indicated in the patient.

It is important to state that thrombophilia screening is not part of routine workup investigations for diagnosing VTE. However, following an acute diagnosis of bilateral PE in our patient, thrombophilia screening was not done immediately, because research evidence shows that thrombophilia screening results are unreliable in the acute phase of an illness such as thrombosis and also during treatment with an oral anticoagulant. Hence, this explains our local hospital policy to refer all patients with newly diagnosed VTE to the anticoagulation clinic for further workup as required by the hematology team.

Furthermore, the absence of significant risk factors for PE and negative D-dimer probably explained why our patient was reassured and discharged to home during her first and second visits, to the ED and EAC, respectively. It is important to know that TXA can alter D-dimer test results, causing false-negative results [14, 16], and this was probably the case during the second hospital visit of our patient, when her D-dimer test result came back negative. A negative D-dimer finding does not completely exclude VTE diagnosis, particularly when there are suggestive clinical histories and signs of VTE [17, 18]. This case report reiterates the need for healthcare practitioners to have a high index of clinical suspicion for PE when patients taking TXA present with pleuritic chest pain and shortness of breath on exertion, particularly in those considered to have low risk for VTE.

Similarly, the available information about the side effects of TXA in the British National Formulary (BNF) describes the risk of embolism or thrombosis as rare or very rare [19]. It is possible the potential risk for developing thromboembolic event while receiving TXA is understated. Recent evidence derived from a retrospective study that investigated the association between TXA and VTE in trauma patients showed that TXA was associated with a greater than threefold increased risk for VTE [20]. Although the odds of developing VTE when taking TXA may be uncommon, it may probably not be as rare or very rare as claimed in the BNF. Some studies that report no differences in thrombotic outcomes in patients taking TXA are probably underpowered to identify thrombotic complications or have short duration of follow-up [21].

Our patient experienced some functional limitations and decreased quality of life with delayed PE diagnosis. She would have continued taking TXA without caution and could have been at risk of developing more serious PE complications, such as chronic thromboembolic pulmonary hypertension, life-threatening acute right (heart) ventricular failure, pulmonary infarction, and cardiac arrest, among other problems [22–24]. Worse still, this woman could have been sent home after the third hospital visit with a false reassurance that her symptoms were likely musculoskeletal pain following the absence of significant risk factors for PE. One can only wonder how many people like our patient might have had a delayed or missed diagnosis of thromboembolic event.

## Conclusion

This case report buttresses previous studies and reinforces the possible association of VTE risk in patients receiving TXA. Although the effectiveness of TXA in treating various medical and surgical conditions cannot be overemphasized, it is imperative that every patient started on TXA be informed of the risk of thromboembolism and be made aware of symptoms suggestive of VTE to enable reporting of symptoms. Also, due to TXA thromboembolic adverse effects, TXA should not be prescribed for patients with significant risk factors for thromboembolic disease, except when the benefit is deemed to outweigh its risk [25]. Further large prospective studies are required to clarify the true risk of VTEs while receiving TXA.

## Data Availability

Not applicable.
